# The complete mitochondrial DNA sequence of endemic honeybee *Apis nuluensis* (Insecta: Hymenoptera: Apidae) inhabiting Mount Kinabalu in Sabah Province, Borneo Island

**DOI:** 10.1080/23802359.2017.1372714

**Published:** 2017-09-05

**Authors:** Jun-Ichi Takahashi, Salim Tingek, Hisashi Okuyama

**Affiliations:** a Department of Life Sciences, Kyoto Sangyo University, Kyoto, Japan;; b Agriculture Research Station, Tenom, Malaysia

**Keywords:** Endemic species, illumina sequencing, Borneo, *Apis nuluensis*, honeybee

## Abstract

The cavity-nesting honeybee *Apis nuluensis* inhabits only the highlands of Mount Kinabalu of Sabah, Borneo Island. The mitochondrial genome is a circular molecule of approximately 1.6 kb that includes 13 protein-coding genes, 22 tRNA genes, two rRNA genes, and one AT-rich control region. The average AT content was 84.5%. The start codons ATC, ATG, and ATT were found in one, three, and nine genes, respectively, whereas the stop codon TAA was observed in all genes. The phylogenetic relationship, inferred using 13 PCGs, was consistent with that reported in previous studies that predicted a sister taxon relationship between *A. nuluensis* and *A. cerana*.

Borneo Island has one of the highest diversities in honeybee species of any region (Koeniger et al. 2010). Recently, an endemic honeybee species inhabiting the highlands of Mount Kinabalu in Sabah Province was discovered and described as a new species, *Apis nuluensis* (Tingek et al. 1996). A morphometric analysis revealed that *A. nuluensis* shows some extreme characteristics that separate it from all other honeybees and it is particularly different from the sympatric species *A. cerana* and *A. koschevnikovi* (Fuchs et al. 1996). Molecular phylogenetic analyses of the partial DNA sequences indicate that *A. nuluensis* is a sister taxon of *A. cerana* with unique haplotypes (Arias et al. 1996; Tanaka et al. 2001; Takahashi et al. 2002; Arias and Sheppard 2005), although one taxonomical study considered *A. nuluensis* to be only a subspecies of *A. cerana* (Engel 1999). Thus, genetic information related to the conservation genetics of *Apis nuluensis*, which is likely an endemic species, is needed. In the present study, we analysed for the first time, to our knowledge, the complete mitochondrial genome of *A. nuluensis* to identify its phylogenetic position and genetic distance from other Asian honeybee species.

We collected adult worker bees of *A. nuluensis* on flowers in the highlands of Crocker Range near Tambunan, Sabah, Malaysia. The collected workers were immediately placed in 95% ethanol for mitochondrial DNA analysis. The specimen was stored in the National Museum of Nature and Science, Japan, accession number: NSMT-I-HYM74241. Genomic DNA was extracted from the thoracic muscle tissue using standard phenol/chloroform methods. Genomic DNA was sequenced using the Illumina’s NextSeq 500 technology (Illumina, San Diego, CA). The complete mitochondrial genomes of the honeybees *A. cerana* (AP018149) were used as reference sequences. The resulting reads were assembled and annotated using the MITOS web server (Bernt et al. 2013) and MEGA6 (Tamura et al. 2013). The phylogenetic analysis was performed using the TREEFINDER v.2011 (Jobb et al. 2004) based on the nucleotide sequences of the 13 protein-coding genes from the complete mitochondrial genome sequences of *Apis* species used in this study, as well as those present in GenBank.

The mitochondrial genome of *A. nuluensis* was a closed loop containing 15,921 bp (AP018157). Similar to the mitochondrial genomes of *A. cerana*, the heavy (H)-strands encoded nine PCGs and 14 tRNA genes whereas the light (L)-strand encoded four PCGs, eight tRNA genes, and two rRNA genes (Takahashi et al. 2016). The average AT content was 84.5%. The genes *ATP8* and *ATP6* shared 19 nucleotides, and *ND4* and *ND4L* shared one nucleotide. The start codon was ATT for the nine PCGs; ATG for *ATP6*, *COIII*, and *Cytb*; and ATC for *ATP8*. The stop codon for all PCGs was TAA. All tRNA genes formed typical cloverleaf secondary structures except for *Ser* (*AGN*).

The phylogenetic analysis suggested that *A. nuluensis* is more closely related to *A. cerana* from Borneo than it is to the other honeybees included in the analysis (Figure 1). This result is consistent with previously published genetic distances and phylogenetic positions inferred from the partial mitochondrial and nuclear sequences of *A. nuluensis* and *A. cerana* (Arias et al. 1996; Tanaka et al. 2001; Arias and Sheppard 2005). This result showed that *A. nuluensis* is not a subspecies or a regional population of *A. cerana*, but a valid species.

**Figure 1.**
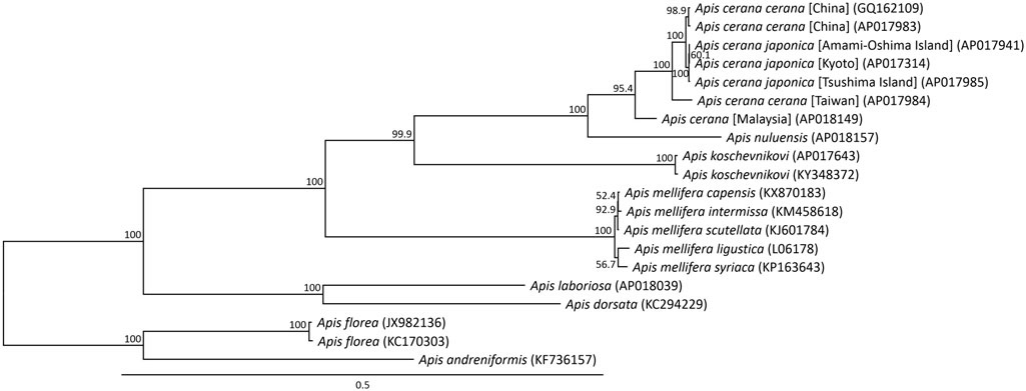
Phylogenetic relationships (maximum likelihood) of species of the genus *Apis* (Hymenoptera) based on nucleotide sequence of 13 protein-coding genes in the mitochondrial genome. Numbers beside each node represent percentages of 1000 bootstrap values. *Apis florea* was used as the outgroup. Alphanumeric terms in parentheses indicate GenBank accession numbers.
